# Effect of Oral Coadministration of Ascorbic Acid with Ling Zhi Preparation on Pharmacokinetics of Ganoderic Acid A in Healthy Male Subjects: A Randomized Crossover Study

**DOI:** 10.1155/2016/2819862

**Published:** 2016-09-25

**Authors:** Patcharanee Tawasri, Chadarat Ampasavate, Somsak Tharatha, Natthakarn Chiranthanut, Supanimit Teekachunhatean

**Affiliations:** ^1^Department of Pharmacology, Faculty of Medicine, Chiang Mai University, Chiang Mai 50200, Thailand; ^2^Department of Pharmaceutical Sciences, Faculty of Pharmacy, Chiang Mai University, Chiang Mai 50200, Thailand; ^3^Central Laboratory (Thailand) Co., Ltd,. Ministry of Agriculture and Cooperatives, Mae Rim, Chiang Mai 50180, Thailand; ^4^Center of Thai Traditional and Complementary Medicine, Faculty of Medicine, Chiang Mai University, Chiang Mai 50200, Thailand

## Abstract

The objective of this randomized, open-label, single-dose, two-phase crossover study was to determine the effect of ascorbic acid on pharmacokinetics of ganoderic acid A, an important biologically active triterpenoid compound with anticancer activities, following oral administration of water extract of fruiting bodies of Ling Zhi in 12 healthy male subjects. Each subject was randomized to receive either one of the two regimens: (1) a single dose of 3,000 mg of the Ling Zhi preparation or (2) a single dose of 3,000 mg of the Ling Zhi preparation in combination with 2,500 mg of ascorbic acid. After a washout period of at least two weeks, subjects were switched to receive the alternate regimen. Blood samples were collected in each phase immediately before dosing and at specific time points for 8 hours after dosing. Plasma ganoderic acid A concentrations were quantified using liquid chromatography-mass spectrometry (LC-MS). The pharmacokinetic parameters analyzed were maximal plasma concentration (*C*_max_), time to reach peak concentration (*T*_max_), area under the plasma concentration-time curve (*AUC*), and half-life (*t*_1/2_). An oral coadministration of ascorbic acid with Ling Zhi preparation did not significantly alter the pharmacokinetic parameters of ganoderic acid A in healthy male subjects.

## 1. Introduction

*Ganoderma lucidum* (Leyss. ex Fr.) Karst., also called Ling Zhi in China and Reishi in Japan, is an oriental mushroom that has been used for thousands of years in East Asia to improve health and longevity [1]. Ling Zhi has also been used to prevent and treat various human diseases including bronchitis, allergies, hepatitis, hypertension, and immunological disorders as well as cancer [1–4]. Oral administration of Ling Zhi extract for 12 weeks has been demonstrated to significantly enhance the immune response in patients with advanced-stage cancers [5]. Additionally, one recent study has found that oral administration of Ling Zhi preparation for 12 weeks seems more capable of stabilizing tumor size compared with a placebo in patients with recurrent gynecologic cancers [6]. Many bioactive components such as polysaccharides and triterpenoids are thought to be associated with the anticancer activities of Ling Zhi [7]. Ganoderic acid A, a highly oxygenated C30 lanostane-type triterpenoid, exhibits significant anticancer activities through inhibition of cancer cell proliferation and metastasis [7, 8]. The inhibition of cancer cell proliferation of ganoderic acid A is believed to be mediated via the downregulation of Cdk4 expression, while its antimetastatic effects appear to be mediated through the inhibition of AP-1/NF-*κ*B-dependent secretion of urokinase plasminogen activator (uPA) [8]. Ganoderic acid A has also been shown to suppress the JAK/STAT3 signaling pathway leading to an enhancement of chemosensitivity to cisplatin in human hepatocellular carcinoma HepG2 cells [9]. Its inhibitory effect on farnesyl protein transferase (FPT), an enzyme that catalyzes the posttranslational farnesylation of the cysteine residue located in the Ras oncoprotein, appears to involve an inhibition of Ras-dependent cell transformation [10].

Ascorbic acid (vitamin C) is an essential micronutrient required for normal metabolic functioning of the human body [11]. It is also an effective water soluble, chain-breaking antioxidant. Recent evidence indicates that an increased intake of ascorbic acid is associated with a reduced risk of chronic diseases such as cancer, cardiovascular diseases, cataracts, and neurodegenerative conditions [11–14]. In addition to its antioxidant capacity [11], ascorbate is a specific cofactor for a large family of enzymes known as the Fe- and 2-oxoglutarate-dependent dioxygenases (2-OGDD) which catalyze the hydroxylation of various substrates. As well as being a well-known cofactor for prolyl hydroxylase, which is essential for the biosynthesis of collagen [15], recent evidence indicates that promotion of the regulatory hydroxylases by higher tumor ascorbate levels can downregulate hypoxia-inducible factor-1 (HIF-1), a transcription factor necessary for maintaining oxygen and energy homeostasis in solid tumors under conditions of cell stress, hence decreasing tumor viability [16]. Epigenetic control in cancer cells mediated via demethylases (which belong to the 2-OGDD family) appears to be affected by higher tumor ascorbate levels [16]. Additionally, high intracellular concentrations of ascorbic acid can act as a prooxidant agent by generating hydrogen peroxide and hence damage the cell membranes of cancer cells. It has also been suggested that the selective toxicity of ascorbic acid in cancer cells is possibly due to reduced levels of catalase, the enzyme that catalyzes the decomposition of hydrogen peroxide into water and oxygen [17–20]. Notably, there is a 10- to 100-fold lower level of catalase in tumor cells than in normal cells [20, 21]. In addition, ascorbic acid exhibits other anticancer effects, possibly by the suppression of angiogenesis, by increasing immunocompetence or by acting as a mitochondrial energy intermediate [22]. High-dose ascorbic acid (10 g/day by continuous intravenous infusion for periods up to ten days and then 2,500 mg/dose orally administered four times a day as a long-term treatment) appears to increase survival, improve well-being, and reduce pain in cancer patients [23–26]. Additionally, recent evidence has shown that intravenous ascorbic acid can improve quality of life and decrease multiple aspects of fatigue in cancer patients [27–32].

A previous pharmacokinetic study has demonstrated that the pharmacokinetic profile of ganoderic acid A following a single oral dose of 3,000 mg of the Muang Ngai 2-strain of Ling Zhi (MG2FB-WE) is characterized by relatively low oral bioavailability and rapid elimination [33]. Although a simple method to enhance the extent of absorbable ganoderic acid A is to increase the ingested Ling Zhi dose, that approach appears to be inappropriate as a relatively high dose of MG2FB-WE has been already administered in this setting. An alternative approach would be to identify agents that would result in an increase in the oral bioavailability of ganoderic acid A when coadministered with Ling Zhi preparations. Among several candidates, ascorbic acid is an agent of interest because several lines of evidence indicate that it may have the potential to enhance the oral bioavailability of several coadministered drugs [34–36]. For example, ascorbic acid enhances the bioavailability of levodopa (a weakly acidic drug with a pKa of 2.3) in elderly patients with Parkinson's disease [34], possibly by increasing the acidity of gastric fluid and intestinal juice, thus enabling more unionized levodopa (a readily absorbable form) to exist in the gastrointestinal tract. Another example is that ascorbic acid enhances reabsorption of furosemide (a weakly acidic drug with a pKa of 3.8) from the renal tubules back into systemic circulation in dogs, presumably through its ability to acidify urine, thus facilitating more of the unionized fraction existing in the tubular lumen to be reabsorbed [35].

Since ganoderic acid A and ascorbic acid exhibit their anticancer activity via the different pathways as mentioned above, coadministration of Ling Zhi preparation with ascorbic acid might show promise as a potential chemotherapeutic intervention in cancer through a synergistic pharmacodynamic effect. Details of the pharmacokinetic interaction between these two interventions are, however, not yet known. The purpose of this study is to determine the effect of ascorbic acid on the pharmacokinetics of ganoderic acid A following oral administration of water extract from the fruiting bodies of Ling Zhi in healthy Thai male subjects. In the present study, we hypothesized that ascorbic acid would increase the bioavailability of ganoderic acid A, a weakly acidic substance with a pKa of 4.35, after oral coadministration of Ling Zhi preparation.

## 2. Materials and Methods

### 2.1. Study Design

The present study was a randomized, open-label, single-dose, two-phase crossover study with a washout period of at least two weeks.

### 2.2. Subjects

Sample size calculation was based on the assumption that the content of absorbed ganoderic acid A (equivalent to the area under the plasma concentration-time curve from time zero to the last measurable sampling time point, *AUC*_0–*t*_) would be the main criterion. The minimum detectable difference in the mean of *AUC*_0–*t*_ between the treatment phases was estimated to be 0.45 ng·h/mL, and the standard deviation of the difference in *AUC*_0–*t*_ for each of the subjects was expected to be 0.50. Using those parameters, the minimum required sample size to achieve an 80% power (or *β* = 0.2) at a 0.05 significance level (*P* value) was calculated to be ten subjects [37].

A total of 12 healthy Thai males aged 18–40 years and with a body mass index (BMI) of 18.5 to 24.9 kg/m^2^ were enrolled in this study. The health of all subjects was assured through a review of their medical history as well as a prestudy physical examination and a laboratory investigation which included a complete blood count, a liver function test, a blood urea nitrogen test, and a creatinine test. All subjects were fully informed about the nature of the study and all signed a written statement regarding their volunteer status prior to the start of the study. Exclusion criteria included hypersensitivity to Ling Zhi or ascorbic acid, chronic renal, liver, neurological, pulmonary, or cardiovascular diseases including malignancy, a personal history of kidney stones, glucose-6-phosphate dehydrogenase deficiency, bleeding disorders, and a family history of iron overload or hemochromatosis. Other exclusion criteria were abuse of alcohol or other substances, cigarette smoking within three months of the study, the use of any Ling Zhi preparations, ascorbic acid, or other medications (with the exception of occasional use of acetaminophen) within one month of the study. Withdrawal criteria for subjects in this study included experiencing adverse effects during the study, an inability to comply with the study protocol, a requirement for other medications during the study period, and voluntary withdrawal from the study. This study was approved by the Human Research Ethics Committee of the Faculty of Medicine, Chiang Mai University, and complied with the Declaration of Helsinki.

### 2.3. Ling Zhi Preparation and Ascorbic Acid

The Ling Zhi preparation used in this study consisted of 3,000 mg of MG2FB-WE prepared in granular formulation which contained 1,417.80 ± 40.74 *μ*g/g of ganoderic acid A (manufactured by the Muang Ngai Special Agricultural Project, Chiang Mai, Thailand, under the patronage of Her Majesty Queen Sirikit). The ascorbic acid used was the commercially available Boots Vitamin C 500® manufactured by Pharmasant Laboratories Co., Ltd., Thailand. Each tablet contained 500 mg of ascorbic acid.

### 2.4. Drug Administration

Subjects were admitted to the Clinical Pharmacology Unit, Faculty of Medicine, Chiang Mai University, after an overnight fast of at least eight hours. Subjects were randomized to receive one of two regimens, either (1) a single dose of 3,000 mg of MG2FB-WE dissolved in 200 mL of warm water (“Ling Zhi” phase) or (2) a single dose of 3,000 mg of MG2FB-WE dissolved in 200 mL of warm water in combination with five tablets of Boots Vitamin C 500 (“Ling Zhi coadministered with ascorbic acid” phase). Subjects remained fasted and upright after drug administration for two and four hours, respectively. Water and lunch were served at two and four hours after dosing, respectively. Blood samples were collected at different time points as described in Section 2.5 below. While waiting for blood sample collection, subjects were allowed to perform daily activities with the exception of moderate to high levels of exercise. After blood sample collection at eight hours after dose, all subjects were discharged from the Clinical Pharmacology Unit.

After a washout period of at least two weeks, subjects were switched to receive the alternate regimen. The blood sample collection and other study conditions in the second study period were the same as in the first study period. Identical food and beverages were served during both study periods. All subjects were instructed to avoid consumption of other Ling Zhi preparations and ascorbic acid throughout the study.

### 2.5. Blood Sample Collection

Serial blood samples (10 mL each) for the determination of the plasma concentration of ganoderic acid A were obtained before oral administration of the Ling Zhi preparation, and at 5, 10, 15, 30, and 45 minutes after dosing then again at 1, 1.5, 2, 2.5, 3, 3.5, 4, 5, 6, and 8 hours after dosing. The blood samples were obtained from the forearm by venipuncture through an indwelling intravenous catheter and collected in heparinized vacutainers. The blood collecting tubes were centrifuged at 1,040 ×g for 15 minutes at 4°C and the plasma was then separated and kept at −70°C until analysis.

### 2.6. Determination of Plasma Ganoderic Acid A by Liquid Chromatography-Mass Spectrometry (LC-MS)

#### 2.6.1. Sample Preparation

The sample preparation for determination of plasma ganoderic acid A concentrations was modified from the method described by Teekachunhatean et al. [33]. An aliquot of plasma (250 *μ*L) was spiked with 25 *μ*L of internal standard (IS, 25 ng/mL of cortisone 21-acetate); 750 *μ*L of 1% acetic acid in 50% methanol/acetonitrile was added for deproteinization. The mixture was vortexed for 30 seconds and then kept at room temperature for 20 minutes. The proteins in the plasma sample were separated by centrifuge at 14,000 ×g for ten minutes at room temperature. Thereafter, an aliquot of the supernatant was isolated and evaporated to dryness using the evaporator at 60°C for 1.5 hours. Residue was reconstituted in 50 *μ*L of the solution consisting of 50% methanol/acetonitrile and 10 mM ammonium acetate buffer (25 : 25 *μ*L), and 20 *μ*L was injected into the LC-MS system. The retention times of ganoderic acid A and IS were 6.31 and 10.08 minutes, respectively. Plasma concentrations of ganoderic acid A were quantified using a calibration curve of the “peak area ratios of ganoderic acid A and IS”* versus* the “respective ganoderic acid A concentrations” and linear regression analysis which consistently provided correlation coefficient values of at least 0.99.

#### 2.6.2. LC-MS Instruments

**Figure 1:**
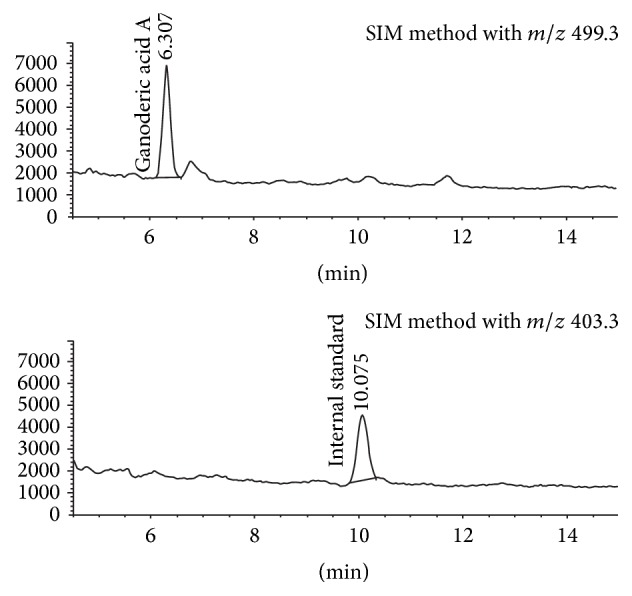
Chromatograms of plasma sample containing 8.00 ng/mL of ganoderic acid A (retention time = 6.31 min) and 2.50 ng/mL of IS (retention time = 10.08 min). The quantitation was performed using the single ion monitoring (SIM) method with mass-to-charge ratio (*m*/*z*) of 499.3 for ganoderic acid A and *m*/*z* of 403.3 for IS.

Determination of ganoderic acid A and IS concentrations was modified from the LC-MS method and conditions described by Teekachunhatean et al. [33]. The assay was carried out using an Agilent Technologies 1100 Series HPLC (Germany). Chromatographic separation was performed with a Zorbax SB-C18 analytical column (150 mm × 4.6 mm, 3.5 *μ*m). The mobile phase consisted of 10 mM ammonium formate buffer with 0.1% formic acid (A) and acetonitrile (B) delivered in a constant ratio of solvent A : B (60 : 40, v/v) at a flow rate of 0.7 mL/min. The column temperature was set at 40°C. The injection volume was 20 *μ*L and the total sample acquisition time was 15 minutes. Mass spectrometric detection was performed on an Agilent Technologies LC/MSD SL Mass Spectrometer in a positive mode. Mass parameters were optimized for the detection: ion spray source temperature at 32°C, nebulizer pressure at 60 psi, capillary voltage at 4000 V, and a gas N_2_ flow rate of 13 L/min. The quantitation was performed using the single ion monitoring (SIM) method with mass-to-charge ratio (*m*/*z*) of 499.3 for ganoderic acid A and *m*/*z* of 403.3 for IS. The chromatograms of plasma sample containing 8.00 ng/mL of ganoderic acid A and 2.50 ng/mL of IS are shown in Figure 1.

#### 2.6.3. Assay Validation

Assay validation was performed following the US Food and Drug Administration guidelines for bioanalytical validation [38]. The lower limit of quantitation (LLOQ) of ganoderic acid A under the LC-MS conditions described above was 0.50 ng/mL. The precision (coefficient of variation) and the accuracy at LLOQ concentration were 3.67 ± 0.02% and 97.90 ± 3.60%, respectively. The intraday precision was 4.92 ± 0.44%, whereas the interday precision was 3.98 ± 0.31%. The intraday and interday accuracy were 101.49 ± 5.01% and 97.97 ± 3.90%, respectively. The mean recovery of ganoderic acid A was 102.15 ± 0.33%.

### 2.7. Pharmacokinetic Parameters

The pharmacokinetic parameters of ganoderic acid A were obtained by noncompartmental analysis of individual plasma concentration-time data using the Topfit software version 2.0. Parameters included maximum plasma concentration (*C*_max_, ng/mL), time taken to reach *C*_max_ (*T*_max_, h), elimination half-life (*t*_1/2_, h), area under the concentration-time curve from time zero to the last measurable sampling time point (*AUC*_0–*t*_, ng·h/mL), and *AUC* from time zero to time infinity (*AUC*_0–*∞*_, ng·h/mL). *C*_max_ and *T*_max_ were determined from observed values, whereas the elimination *t*_1/2_ was determined from the ratio of 0.693/*Ke*, where *Ke* was the elimination rate constant. *AUC*_0–*t*_ was calculated using a trapezoidal rule. The extrapolated *AUC* from time *t* to infinity (*AUC*_*t*–*∞*_) was calculated as *Ct*/*Ke* and *AUC*_0–*∞*_ was the summation of *AUC*_0–*t*_ + *AUC*_*t*–*∞*_.

### 2.8. Statistical Analysis

Since the sample size used in this study was small and the distribution of the measured parameters could not be assumed to be approximately normally distributed, the differences in the mean values of each of the pharmacokinetic parameters between the two treatment phases (Ling Zhi phase* versus* Ling Zhi coadministered with ascorbic acid phase) were compared using the nonparametric Wilcoxon signed-rank test. *P* values < 0.05 were considered to be statistically significant.

## 3. Results

The mean values (±standard deviation) of age, weight, height, and BMI of the 12 subjects enrolled in this study were 24.33 ± 2.35 years, 64.88 ± 6.47 kg, 1.69 ± 0.06 m, and 22.79 ± 1.43 kg/m^2^, respectively. All completed the study protocol without any adverse events. In the determination of individual pharmacokinetic parameters, it was found that the plasma concentration-time curve of Ling Zhi phase could not be plotted for one subject because his ganoderic acid A levels at all time points (except at 0.75 hours) were below LLOQ, leading to an inability to calculate *t*_1/2_, *AUC*_0–*t*_, and *AUC*_0–*∞*_ using the Topfit software. However, the plasma concentration observed at 0.75 hours in this subject provided a value for his *C*_max_ and *T*_max_. Therefore, the sample size used for comparison of the mean values of *C*_max_ and *T*_max_ between the two treatment phases was 12 subjects, whereas only 11 subjects were included in the comparison of the remaining pharmacokinetic parameters.

**Figure 2:**
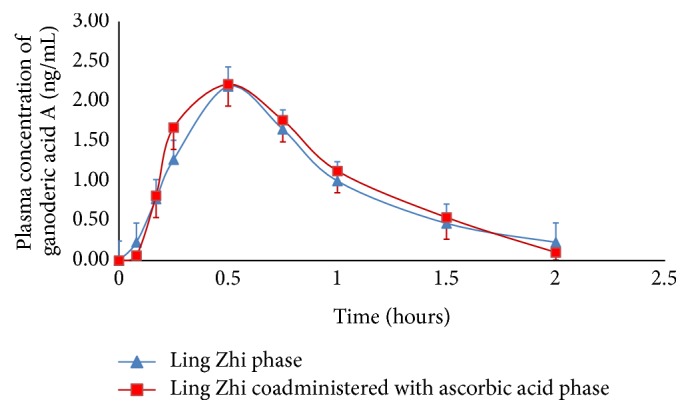
Mean plasma ganoderic acid A concentration-time curves after a single oral dose of 3,000 mg of MG2FB-WE alone (Ling Zhi phase) and in combination with 2,500 mg of ascorbic acid (Ling Zhi coadministered with ascorbic acid phase) in healthy male subjects. MG2FB-WE refers to water extract of fruiting bodies of the Muang Ngai 2-strain Ling Zhi in granular formulation. Error bars represent standard deviations (SD).

The mean plasma ganoderic acid A concentration-time curves obtained from Ling Zhi phase and Ling Zhi coadministered with ascorbic acid phase are shown in Figure 2. The pharmacokinetic parameters of ganoderic acid A obtained from both phases are provided in Table 1. An oral coadministration of 2,500 mg of ascorbic acid with 3,000 mg of MG2FB-WE Ling Zhi preparation did not significantly affect *C*_max_, *T*_max_, *AUC*_0–*t*_, *AUC*_0–*∞*_, or *t*_1/2_ of ganoderic acid A in the healthy male subjects.

## 4. Discussion

This randomized, two-phase crossover study found that oral coadministration of ascorbic acid with MG2FB-WE Ling Zhi preparation in healthy male subjects did not significantly alter the pharmacokinetic parameters of ganoderic acid A, an important biologically active triterpenoid compound with well-known anticancer activities found in Ling Zhi. A crossover design was considered an appropriate choice because each crossover patient served as his own control; therefore, intersubject variability between the two treatment phases could be minimized. The study was conducted under fasted conditions because a previous study by the authors had demonstrated that intake of food significantly reduces *C*_max_ and delays *T*_max_ of ganoderic acid A, although it does not affect the extent of its absorption [33].

The dose of 3,000 mg of MG2FB-WE Ling Zhi preparation was chosen for evaluating herb-drug interaction in the present study because a previous randomized double-blind study found that oral administration of 3,000 mg/dose of MG2FB-WE twice daily for 12 weeks seems more capable of stabilizing tumor size compared with a placebo in patients with gynecologic cancers after failure of at least two regimens of conventional chemotherapy [6, 39]. The dose of ascorbic acid investigated in this study was chosen based on previously reported data which suggested that a continuous oral administration of 2,500 mg/dose four times a day may have a palliative effect in the treatment of terminal human cancer [23–26].

The present pharmacokinetic study, performed under fasted conditions, found that ganoderic acid A had a rapid *T*_max_ of 0.54 ± 0.10 hours and a short elimination *t*_1/2_ of 0.66 ± 0.33 hours. These pharmacokinetic parameters are in accordance with the values of 0.54 ± 0.18 hours and 0.62 ± 0.17 hours, respectively, reported in our previous study [33]. Investigation of herb-drug interaction in the present study found that oral coadministration of ascorbic acid with Ling Zhi preparation did not significantly affect *C*_max_, *T*_max_, *AUC*_0–*t*_, *AUC*_0–*∞*_, or *t*_1/2_ of ganoderic acid A. That finding could be interpreted as meaning simply that ascorbic acid does not cause significant changes in the rate and extent of ganoderic acid A absorption or in the elimination rate of ganoderic acid A. This finding contradicts our hypothesis that ascorbic acid would increase the bioavailability of ganoderic acid A, a weakly acidic substance with a pKa of 4.35, after oral coadministration of Ling Zhi preparation. This discrepancy might be due to the basal pH of gastric juice in our subjects under fasted conditions being already low enough to enable nearly the maximum potential concentration of unionized ganoderic acid A (an absorbable form) to exist in the gastrointestinal tract; therefore, coadministration of ascorbic acid under these conditions might not provide further reduction in gastric pH and thus not affect the rate or extent of ganoderic acid A absorption.

It can be assumed that although ascorbic acid did not enhance the oral bioavailability of ganoderic acid A, the acceptable safety profiles from long-term treatment of either Ling Zhi preparation or high-dose ascorbic acid [40, 41] and the possibility that this combination might provide a synergistic pharmacodynamic effect in cancer treatment suggest that this combination is a potential option for cancer patients, especially in the final stages of terminal cancer; however, this option should be carefully considered and recommended to patients at the physician's discretion. Nevertheless, the potential synergy from a combination of Ling Zhi preparations and ascorbic acid in cancer patients warrants further intensive clinical investigations.

Some major limitations in the present study should be addressed. First, this study aimed to investigate the effects of a single dose of ascorbic acid orally coadministered with Ling Zhi preparation; the coadministration of both medications in multiple dosing regimens (repetitive dosing) and determination of their pharmacokinetic interaction at steady state should be further investigated. Second, the current study focused on the influence of oral coadministration of ascorbic acid with Ling Zhi preparation on the pharmacokinetics of ganoderic acid A but did not investigate possible impacts on other biologically active compounds in the Ling Zhi preparation; in addition, it did not answer the question of whether Ling Zhi preparation affects the pharmacokinetics of ascorbic acid. Third, the antioxidant capacity of ascorbic acid, as well as its effects on hypoxia-inducible factor regulation and epigenetic control, should be further investigated, especially following the long-term coadministration with Ling Zhi preparation in cancer patients. Fourth, although blood samples used to determine plasma ganoderic acid A concentrations were serially collected until eight hours after dosing in order to obtain adequate detectable values during the elimination phase, in practice plasma concentrations in all subjects were quantifiable for a maximum of two hours after dosing because the sensitivity of LC-MS used for detecting plasma ganoderic acid A concentration in this study was limited to an LLOQ of 0.50 ng/mL. A more sensitive quantitative bioanalysis should be considered in further studies; however, a recent study demonstrated that detection of plasma ganoderic acid A concentrations using LC-MS-MS provides a sensitivity (LLOQ) that is not greater than that of LC-MS [42]. Finally, although the 12 subjects enrolled in this study appear to be a rather small sample, post hoc analysis based on the data from the present study demonstrates a power of test (1-*β*) of 87% (based on an estimated minimum detectable difference in means of *AUC*_0–*t*_ between the treatment phases of approximately 25%), indicating acceptable statistical power.

## 5. Conclusions

An oral coadministration of ascorbic acid with MG2FB-WE Ling Zhi preparation did not significantly alter pharmacokinetic parameters of ganoderic acid A (i.e., *C*_max_, *T*_max_, *AUC*_0–*t*_, *AUC*_0–*∞*_, and *t*_1/2_) in healthy male subjects.

## Figures and Tables

**Table 1:** Pharmacokinetic parameters of ganoderic acid A following a single oral dose of 3,000 mg of MG2FB-WE alone (Ling Zhi phase) and in combination with 2,500 mg of ascorbic acid (Ling Zhi coadministered with ascorbic acid phase) in healthy male subjects.

Pharmacokinetic parameter	Ling Zhi phase	Ling Zhi coadministered with ascorbic acid phase	*P* value^*∗*^
*C*_max_ (ng/mL)	2.24 ± 1.31	2.39 ± 0.99	0.41
*T*_max_ (h)	0.54 ± 0.10	0.44 ± 0.19	0.10
*AUC*_0–*t*_ (ng·h/mL)	1.93 ± 0.96	2.04 ± 0.74	0.53
*AUC*_0–*∞*_ (ng·h/mL)	2.71 ± 1.09	2.82 ± 0.88	0.79
*t*_1/2_ (h)	0.66 ± 0.33	0.66 ± 0.30	0.86

Data represent mean ± standard deviation (SD). *C*_max_ = maximum plasma concentration. *T*_max_ = time taken to reach *C*_max_. *AUC*_0–*t*_ = area under the concentration-time curve from time zero to the last measurable sampling time point. *AUC*_0–*∞*_ = area under the concentration-time curve from time zero to time infinity. *t*_1/2_ = elimination half-life. ^*∗*^Wilcoxon signed-rank test.
